# Oncolytic Viruses Partner With T-Cell Therapy for Solid Tumor Treatment

**DOI:** 10.3389/fimmu.2018.02103

**Published:** 2018-09-21

**Authors:** Amanda Rosewell Shaw, Masataka Suzuki

**Affiliations:** ^1^Department of Medicine, Baylor College of Medicine, Houston, TX, United States; ^2^Baylor College of Medicine, Center for Cell and Gene Therapy, Texas Children's Hospital, Houston Methodist Hospital, Houston, TX, United States

**Keywords:** oncolytic virus, CAR-T cell, bispecific T-cell engager (BiTE), cytokine, chemokine, checkpoint inhibitor

## Abstract

Adoptive T-cell immunotherapies, including chimeric antigen receptor-modified T-cells (CAR-T cells), have revolutionized cancer treatment, especially for hematologic malignancies. Clinical success of CAR-T cell monotherapy in solid tumors however, has been only modest. Oncolytic viruses provide direct cancer cell lysis, stimulate systemic immune responses, and have the capacity to provide therapeutic transgenes. Oncolytic virotherapy has shown great promise in many preclinical solid tumor models and the first oncolytic virus has been approved by the FDA for the treatment of advanced melanoma. As monotherapies for solid tumors, oncolytic virotherapy provides only moderate anti-tumor effects. However, due to their complementary modes of action, oncolytic virus and T-cell therapies can be combined to overcome the inherent limitations of each agent. This review focuses on the aspects of oncolytic viruses that enable them to synergize with adoptive T-cell immunotherapies to enhance anti-tumor effects for solid tumors.

## Introduction

Clinical use of adoptive cell therapies to treat cancer has gained great interest in recent years, adding new treatment options to the paradigm of surgery, radiotherapy, and chemotherapy. Bolstering a patient's immune system with infused T-cells that have been genetically modified to specifically target tumor cells holds great promise and has demonstrated clinical efficacy in hematologic malignancies (1). These T-cells are genetically modified to express a chimeric antigen receptor (CAR) with an extracellular domain derived from single chain variable fragment (scFv) specific to a target surface antigen on cancer cells and intracellular CD3ζ signaling domain. CARs can be further modified to include co-stimulatory domains like CD28, 4-1BB and ICOS, resulting in a cell that can respond to tumor antigens by proliferating and killing target cells dependent upon target antigen expression (CAR-T cells) (2).

**Figure 1:**
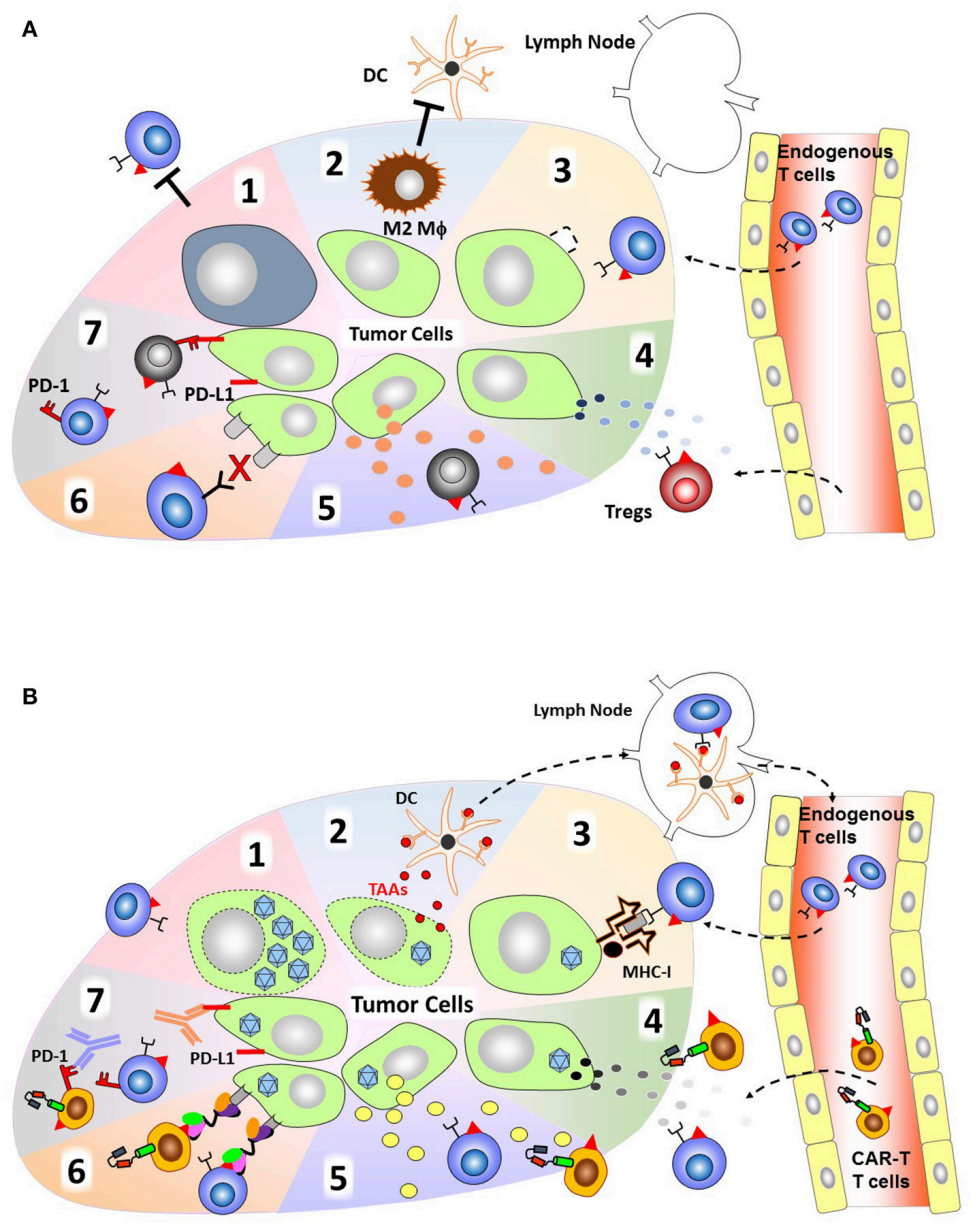
Attributes of OVs to overcome immunosuppression by the tumor microenvironment. **(A)** The immunosuppressive tumor microenvironment. (1) T-cells have poor accessibility to dense, bulky tumors. (2) Presence of immunosuppressive cells such as myeloid derived suppressor cells (MDSCs) and M2 macrophages. (3) Downregulated MHC-I expression resulting in poor antigen presentation/recognition. (4) Tumor cells secrete chemokines attract immunosuppressive cells such as regulatory T-cells (Tregs). (5) Tumor cells can also secrete inhibitory cytokines (e.g., TGF-β, IL-(10) that inhibit cytotoxic T-cell function. (6) Cancer cells often lack tumor specific antigens that can be recognized by endogenous T-cells. (7) Expression of immune checkpoint molecules (e.g., PD-L1) that cause exhaustion upon engagement of cognate receptors on T-cells (e.g., PD-1). **(B)** Mechanisms by which oncolytic viruses can help T-cells to overcome the immunosuppressive environment. (1) Direct oncolysis of tumor cells and increased tumor accessibility by creating space within the tumor mass. (2) Release of DAMPs, PAMPs, and TAAs upon tumor cell lysis that can recruit APCs, and TAAs can be processed and presented to T-cells at lymph node. (3) OV infection can induce expression of MHC-I and β2M. (4) OVs can be engineered to express chemokines to increase infiltration of both endogenous T-cell and CAR T-cell. (5) Express inflammatory cytokines to increase T-cell proliferation at the tumor site. (6) Produce BiTE (Engager) molecules to redirect T-cells to tumor specific antigens. (7) Express Checkpoint inhibitors for attenuating T-cell exhaustion.

Adoptive cell transfer of autologous CAR-T cells targeting B-cell antigen CD19 have resulted in profound remission in patients with refractory B-cell malignancies. Recently, the first chimeric antigen receptor CAR-T cells, Tisagenlecleucel (3), have been approved by the FDA for the treatment of acute lymphoblastic leukemia. Followed in quick succession by the approval of a second CAR-T cell therapy, Axicabtagene ciloleucel for lymphomas (4). However, the use of adoptive cell therapies for the treatment of solid tumors as a monotherapy has been less successful. Compared to hematological malignancies, clinical outcome in trials utilizing CAR-T cells to target various solid tumors has a much higher rate of patients achieving only stable disease and no response/progressive disease (5). The major barriers to successful CAR-T cell therapies for solid tumors include; lack of tumor specific or downregulation of antigen expression, the immunosuppressive tumor microenvironment which lacks necessary pro-inflammatory stimulatory molecules and is abundant with inhibitory checkpoint molecules, and physical barriers of the solid tumor mass (6) (Figure 1A). These preclinical and clinical trials suggest that CAR-T cells are insufficient to overcome these inhibitory mechanisms as a monotherapy and therefore require additional therapy to enhance their anti-tumor effect.

Oncolytic viruses (OVs) have been designed to selectively replicate in and kill cancer cells. It is well established that OVs can stimulate adaptive immune responses to tumor cells due to the release of tumor associated antigens (TAAs), pathogen-associated molecular patterns (PAMPS), and danger-associated molecular patters (DAMPS) from lysed tumor cells. These responses also shift tumors from cold (immune desert) to hot (inflamed) tumors (7). Once processed by antigen presenting cells (APCs), TAAs can then induce anti-tumor T-cell responses in parallel with anti-viral responses. Based on these unique features, OVs are now considered a cancer immunotherapy agent (7). However, OV treatment alone is still unable to cure bulky and/or metastasized tumors and thus OVs also require additional therapies to enhance their anti-tumor effect. OVs have the added advantage of being able to deliver therapeutic transgenes to further enhance anti-tumor activity of host immune cells (“Armed” OVs; Figure 1B). Although the anti-tumor capacity of OVs including “Armed” OVs has been investigated for decades, OVs are only now being used clinically after the recent approval of talimogene laherparepvec (T-VEC), a herpes simplex-1 (HSV) oncolytic virus expressing granulocyte macrophage colony-stimulating factor (GM-CSF), for the treatment of malignant melanoma (8). Intralesional administration of T-VEC induces a systemic immune response as indicated by reduction in size of untreated lesions (abscopal effect). However, clearance of these lesions was incomplete. Combining T-VEC with immune checkpoint inhibition provides complementary immune stimulation mechanisms as demonstrated in recent case studies (9–11). These clinical results clearly indicate that combination of OVs with another immunotherapy agent has additive anti-tumor effects. Combining OVs with CAR-T cell treatment strategies could function in a complementary and additive manner by overcoming the limitations of each treatment moiety (e.g., limited anti-tumor effects of OV to distant (untreated) sites, limited accessibility/persistence of CAR-T cells at tumor sites). This review will focus on recent developments and applications in OVs that have the potential to synergize with adoptive T-cell immunotherapy.

## Combination of OVs with adoptive T cell therapy

Combinatorial treatment with OVs has been demonstrated to augment the anti-tumor activity of adoptively transferred T-cells (12). In a syngeneic immunocompetent mouse model using B16ova melanoma, it was demonstrated that intratumoral administration of oncolytic vesicular stomatitis virus (oVSV) leads to increased CD8^+^ T cell infiltration and resulted in 50% survival within 30 days, compared to treatment with heat-inactivated oVSV or mice left untreated whose median survival was approximately 20 days. Similarly, infusion of OT-I (OVA-specific) T-cells resulted in 50% survival within 30 days. To enhance the antitumor effect, the authors combined oVSV treatment with systemic infusion of OT-I T-cells resulting in a more potent anti-tumor response than either single agent treatment, approximately 70% survival at 50 days (13). In a similar model, intratumoral administration of oncolytic adenovirus combined with *ex vivo* activated OT-I T-cells led to increased presence of endogenous CD8^+^ T-cells resulting in rejection of tumor re-challenge (14). Thus, combining oncolytic virotherapy with adoptive T-cell immunotherapy has proven to be beneficial in immunocompetent mouse models. These results suggest that OVs and T-cell therapy independently and additively function to control tumor growth.

### OVs for T cell retargeting

One anti-tumor T-cell mechanism relies on the ability of the T-cell to recognize tumor antigens, thereby priming the T-cell to produce a cytolytic effect. Unfortunately, tumor cells are adept at escaping immune surveillance. One mechanism for this escape is the dysfunctional antigen processing of tumor cells through reduced expression of the major histocompatibility complex class I (MHC-I) (15). In heterogeneous solid tumors a tenuous balance is struck in which cytotoxic T-cells can eliminate the most susceptible tumor cells with high expression of target antigens. However, tumors can undergo a process of immune editing by which tumor cells that rapidly divide have increased mutational burden leading to downregulation or loss of target antigens. Once the infiltrated T-cells kill the tumor cells expressing a target antigen the remaining cancer cells can no longer be targeted by the T-cells, resulting in tumor immune escape and outgrowth (16). Even in hematologic malignancy, although CD19 is expressed on essentially all cases of B-cell Acute Lymphoid Leukemia (B-ALL) at clinical presentation, relapses with loss or diminished surface expression of CD19 are increasingly recognized as a cause of CD19.CAR-T cell treatment failure (17). Other clinical data has suggested that T-cell based immunotherapy leads to downregulation of MHC-I through loss of functional β2-microglobulin (18). An advantage of OVs is that MHC expression is induced after OV infection of cancer cells as demonstrated by oncolytic herpes simplex virus (19). Additionally, measles virus induces MHC and costimulatory molecules (20), and reovirus induces MHC-I as well as β2-microglobulin, TAP-1, and TAP-2 to enhance antigen presentation (21, 22). The potential of oncolytic virotherapy to overcome the attenuation of antigen escape induced by T-cell immunotherapy is a benefit of combination therapy.

Bispecific T cell engagers (BiTEs) are molecules consisting of a CD3-scFv linked to another scFv specific for an antigen expressed on the surface of tumor cells. By utilizing these molecules, tumor resident/infiltrated T-cells can be redirected toward additional specific antigens expressed on cancer cells. Blinatumomab is an FDA approved CD19 BiTE for the treatment of relapsed or refractory B-ALL (23) which functions to educate cytotoxic T cells to target malignant B-cells expressing CD19 (24). In a phase III trial comparing Blinatumamab to standard chemotherapy, complete remission rates (34 vs. 16%) and overall survival (7.7 vs. 4 months) were significantly improved in patients receiving the BiTE. However, due to the short half-life of the BiTE molecule, the drug must be administered by continuous infusion and the vast majority of patients (87%) receiving Blinatumamb had grade 3 or higher adverse events (25). Although there are currently many BiTE molecules in development for clinical use (26), this potential side effect due to systemic and frequent infusion may need to be addressed.

To increase the efficacy of BiTE molecules and decrease unwanted side effects due to constant systemic administration, local constitutive expression of BiTEs at the tumor site would provide stimulation for tumor resident T-cells without systemic toxicity. To this end, OVs have been used to express various BiTE molecules, providing a retargeting moiety to T-cells together with virus mediated oncolysis. To target tumor cells expressing the EphA2 antigen, an oncolytic vaccinia virus (VV) was engineered to express an EphA2 BiTE, called T-cell engager armed VV (TEA-VV). In an orthotopic lung tumor xenograft model, when human PBMCs were delivered together with the EphA2.TEA-VV, tumor growth was significantly reduced compared to mice receiving only oncolytic VV or unarmed oncolytic VV with PBMCs (27).

Likewise, an oncolytic adenovirus (Onc.Ad) expressing an EGFR-BiTE (Onc.Ad-EGFR.BiTE), derived from cetuximab which is used clinically to treat colorectal (28) and head-and-neck squamous cell carcinomas (29), was able to induce *ex vivo* activated, adoptively transferred T-cell accumulation and proliferation in a subcutaneous model of colorectal carcinoma. Administration of unarmed Onc.Ad provided oncolysis and reduced tumor growth which was significantly enhanced by the addition of the BiTE molecule in the presence of activated T-cells (30). However, this Onc.Ad-EGFR.BiTE combined with transferred unstimulated T-cells required systemic administration of IL-2 and did not clear the tumors, suggesting that additional activation and/or persistence of T-cells at the tumor site is required to lead T-cell dependent anti-tumor effect through the BiTE molecule. The group then tested their Onc.Ad-EGFR.BiTE combined with CAR-T cells targeting another antigen, folate receptor alpha (FR-α) which had been previously tested and shown to be safe but not efficacious in patients with metastatic ovarian cancer (31). Treatment with Onc.Ad-EGFR.BiTE was able to increase FR.CAR-T cell killing, proliferation, and IFNγ production *in vitro*. *In vivo*, Onc.Ad-EGFR.BiTE combined with two administrations of FR.CAR-T cells significantly delayed tumor growth in a xenograft model in which the tumor cells expressed intermediate levels of FR-α and high levels of EGFR. In a second *in vivo* model, the tumor cells expressed low levels of FR-α, and high levels of EGFR, the combination of the Onc.Ad with CAR-T cells resulted in sustained reduction of tumor volume compared to single agent treatments. Additionally, when the Onc.Ad-EGFR.BiTE was combined with an irrelevant CAR-T cell, the presence of the BiTE molecule increased CAR-T cell infiltration and activation markers similar to the FR.CAR-T treatment (32) (Table 1). Thus, demonstrating the combination of viral-mediated oncolysis with retargeting of immune cells to secondary targets can produce an additive anti-tumor effect of CAR-T cells.

**Table 1:** Preclinical studies combining oncolytic viruses with CAR-T cells.

**Virus**	**Tumor**	**CAR antigen**	**CAR endodomain**	**Dose/mouse**	**References**
Onc.Ad-EGFR BiTE	Pancreatic ductal carcinoma/colorectal carcinoma	Folate receptor alpha (FR-α)	41BB	1 × 10^7^ CAR-T 1 × 10^9^ Onc.Ad/ 1 × 10^7^ CAR-T (2x) 1 × 10^9^ Onc.Ad	(32)
Onc.Ad-TNFα/IL2	Pancreatic ductal carcinoma	Mesothelin (meso)	41BB	1 × 10^6^ CAR-T 3 × 10^9^ Onc.Ad (xenograft)/5 × 10^6^ CAR-T 1 × 10^9^ Onc.Ad (syngeneic)	(33)
Onc.Ad-Rantes/IL15	Neuroblastoma	Ganglioside GD2	CD28 &OX40	1 × 10^7^ CAR-T 1 × 10^6^- 1 × 10^9^ Onc.Ad	(34)
CAdVEC-αPDL1	Prostate, Squamous Cell Carcinoma	Human epidermal growth factor 2 (HER2)	CD28	1 × 10^6^ CAR-T 1 × 10^7^ Onc.Ad	(35)
CAdVEC-IL12p70/αPDL1	Head and neck squamous cell carcinoma	Human epidermal growth factor 2 (HER2)	CD28	1 × 10^6^ CAR-T 1 × 10^8^ Onc.Ad	(36)

### OVs expressing cytokine/chemokine

Before T cells can perform their cytotoxic functions at tumor sites, they must first home to their target and infiltrate the tumor mass. Chemokines are molecules that serve to draw immune cells to sites of inflammation. It was recently demonstrated that intratumoral administration of an oncolytic type II herpes simplex virus (HSV-2) induces high expression of multiple pro-inflammatory chemokines (i.e., CCL2, CCL3, CCL4, CXCL9, CXCL10, CXCL11) resulting in increased accumulation and persistence of adoptively transferred OT-I T-cells in both immune competent and incompetent models (37).

The suppressive tumor microenvironment is depleted of pro-T cell cytokines which is a significant inhibitory mechanism tumors develop to evade cytotoxic T-cells. OVs can deliver molecules to stimulate T-cells at the tumor site and reverse this anergy. Administration of an Onc.Ad expressing TNFa and IL-2 (Onc.Ad-IL2/TNFa) in five consecutive doses has significant antitumor effect in an immune competent Syrian hamster model of pancreatic cancer (38). Subsequently, this Onc.Ad-IL2/TNFa was combined with mesothelin-CAR-T cells (meso.CAR-T), tested in patients with pancreatic adenocarcinoma or mesothelioma (39), in a preclinical model of pancreatic ductal adenocarcinoma. A single intratumoral administration of the Onc.Ad followed 3 days later with systemic administration of meso.CAR-T resulted in 100% survival after 100 days compared to median survival of 56 days in mice treated with only meso.CAR-T or Onc.Ad-IL2/TNFa. Importantly, the combination of Onc.Ad-IL2/TNFa with meso.CAR-T was able to inhibit the formation of lung metastases. In a syngeneic immunocompetent model, mice treated with murine meso.CAR-T had little tumor control but when combined with non-replicating Ad vectors expressing murine IL-2 and TNFa complete short-term tumor inhibition was achieved. The Ad vectors themselves or combined with an irrelevant CAR-T cell, lead to host immune cell infiltration and caused a reduction in tumor volume, demonstrating the benefit of activating host immune responses for combinatorial treatment (33).

To increase the efficiency of CAR-T cell trafficking and persistence within tumors, production of proinflammatory chemokines and cytokines from the tumor mass has been investigated. An Onc.Ad expressing both IL-15 and RANTES (Onc.Ad-IL15/RANTES) has demonstrated that combining both molecules can have a profound effect on adoptively transferred GD2.CAR-T cells, which have accomplished remission in patients with neuroblastoma (40). Intratumor administration of Onc.Ad-IL15/RANTES increased the infiltration and persistence of GD2.CAR-T cells in a xenograft model of neuroblastoma resulting in significantly enhanced survival (34). This work establishes the potential of utilizing oncolytic viruses armed with proinflammatory molecules to increase the antitumor activity of CAR-T cells that have modest effects on their own.

### OVs and checkpoint blockade

One of the strongest barriers to successful T-cell therapy for solid tumors is the expression of inhibitory immune checkpoint ligands expressed on tumor cells (41) (Figure 1A). These ligands shut down effector T-cell function resulting in their inability to attack and control cancer cells. Antibodies targeting these immune checkpoint molecules can be effective in reversing this T-cell hypofunction which is reflected in the recent, rapid approval of these antibodies for clinical use. These antibodies, however, are associated with systemic toxicities and are only modestly efficacious as monotherapies (42).

Cancer cells upregulate the T-cell inhibitory ligand PD-L1 in the presence of IFNγ, which is produced by activated T cells, and CAR-T cells express PD-1 upon activation (35). Our group has recently demonstrated that a combinatorial Ad vector expressing a PD-L1 blocking mini-antibody (CAdVEC*PDL1*) enhances the antitumor effect of HER2.CAR-T cells, which were recently reported to be safe in patients with sarcoma (43), against multiple human cancer cells *in vitro* and *in vivo* (35). Local expression of the PD-L1 blocking antibody via CAdVEC*PDL1* treatment proved to be less toxic and provide greater tumor control than systemic administration of PD-L1 antibody. While this combinatorial treatment strategy is effective, providing significant long-term survival advantage, it was not curative in subcutaneous xenograft models. We then utilized our adenoviral vector to deliver a stimulatory cytokine in addition to the PD-L1 blocking antibody since there is ample evidence of oncolytic vectors expressing cytokines enhancing adoptive T-cell therapies as described above. We generated a library of helper-dependent Ads expressing various cytokines (IL2, IL7, IL-12p70, IL15, and IL21) and screened them for their ability to enhance HER2.CAR-T mediated killing of head-and-neck squamous cell carcinoma (HNSCC) targets. We found that only IL-12p70 mediated tumor regression in conjunction with PD-L1 blocking antibody in both HPV positive and negative HNSCC xenograft models. We then generated a single vector expressing IL-12p70 with the PD-L1 blocking antibody which was co-injected with an oncolytic adenoviral vector (CAdVEC*IL12_PDL1*). This treatment combined with HER2.CAR-T cells was able to control both primary and metastasized tumors in an orthotopic model of HNSCC causing lymph node metastasis similar to those seen in HNSCC patients. This superior anti-tumor effect leads to 100% survival of animals treated with the combination of CAR-T cells and CAdVEC*IL12_PDL1* for more than 120 days without xenogenic GVHD after single treatment (36). These results suggest that OVs expressing a checkpoint inhibitor in conjunction with CAR-T cell treatment is effective, but CAR-T cells require additional signals (e.g. cytokine) to maintain anti-tumor effects. We expect that only blockade of PD-1:PD-L1 interaction may lead to over-activation of CAR-T cells and results in immediate exhaustion, but provision of appropriate cytokine (Signal 3) can attenuate this exhaustion. However, the mechanism by which exogenous IL-12p70 (STAT4 activation) leads to long-term anti-tumor effect of CAR-T cells and how blockade of PD-1:PD-L1 interaction contributes to IL-12p70 signaling is still unclear.

## Conclusion

These preclinical data clearly demonstrate that, although tumors are adept at evading immunotherapies, combining OVs with adoptive T-cell immunotherapeutic strategies can overcome these evasion mechanisms. Based on previous clinical trials with mono-immunotherapy, combining immunotherapy regimens that target different aspects will be necessary to eradicate tumors. OVs provide complementary antitumor mechanisms such as stimulation of innate immune responses, increasing tumor antigen presentation, and direct oncolysis of tumors. Additionally, OVs can provide targeting molecules like bispecific T-cell engagers, stimulatory cytokines, chemokines, and even immune checkpoint inhibitors. However, most preclinical studies combining OVs and CAR-T cells are based on immunodeficient mouse models, and further investigation using immunocompetent models (e.g., humanized mouse) will be needed to understand how host immune responses (e.g., anti-viral response) contribute to this combinatorial therapy.

Armed OVs can be rationally designed to provide T-cells with optimal synergistic molecules for specific tumor targets and therefore represent an ideal platform for targeted cancer therapies. Based on clinical trial data with CAR-T cells for solid tumors, we may be able to identify appropriate molecule(s) expressed by OVs for each CAR construct and for each target tumor/tissue to maximize the anti-tumor effect of CAR-T cells. Since safety of both agents as monotherapy have been demonstrated in numerous clinical trials, and our data indicate that we can obtain durable responses with 1-2 log lower dosages of each agent used as monotherapy in preclinical models (35, 36), combination of OVs and CAR-T cell therapy may be a safer and more effective treatment in future clinical trials for solid tumors.

## Author contributions

All authors listed have made a substantial, direct and intellectual contribution to the work, and approved it for publication.

### Conflict of interest statement

The authors declare that the research was conducted in the absence of any commercial or financial relationships that could be construed as a potential conflict of interest.
